# Women’s Engagement With Different Internet-Enabled Technologies to Access Digital Menopause Information: Mixed Methods, Multiphase Sequential Study

**DOI:** 10.2196/78215

**Published:** 2026-05-20

**Authors:** Alison K Osborne, Elizabeth Sillence, Caroline Claisse, Abigail C Durrant

**Affiliations:** 1 Department of Psychology, Faculty of Health and Life Sciences, Northumbria University, Northumberland Building, Newcastle upon Tyne, NE1 8ST, United Kingdom,; 2 School of Psychology, Faculty of Health and Wellbeing, Northumbria University, Newcastle upon Tyne, NE1 8ST, United Kingdom, 44 191243 7246; 3 Open Lab, School of Computing, Newcastle University, Newcastle upon Tyne, United Kingdom

**Keywords:** menopause, digital health, women’s health, health communication, digital technology, websites, podcasts, online forums, health information seeking behavior, internet, mixed methods research, qualitative research

## Abstract

**Background:**

Information on menopause can come from a variety of sources, from friends and family to health care professionals, but increasingly, digital information has become a significant source. Digital information can be accessed online, through websites, social media, podcasts, and online groups or forums. The extent to which digital information on menopause is accessed and consumed can vary widely depending on individual circumstances. Existing literature has focused on investigating a single technology at a time in terms of digital menopause information, rather than exploring a wider ecosystem that people may use. As a result, existing evidence is perhaps limited in the understanding of the role these internet-enabled digital technologies play holistically, specifically for digital menopause information.

**Objective:**

This study explored how women engage with different internet-enabled technologies to access digital menopause information and to understand how the technologies relate to feelings of competence, autonomy, and relatedness. Using a feminist new materialism lens, participants’ experiences of different technologies are considered in terms of the affordances of the technology, the associated affective factors, and the agential capacities created.

**Methods:**

Sixteen women aged between 40 and 62 years, who were going through the menopause, took part in a multiphase, mixed methods study. Initially, participants completed an online survey to capture demographics, their perceived knowledge of menopause, and information regarding their existing digital engagement. This was followed by an entry interview to establish the context of their experiences. Participants then completed 3 menopause information gathering tasks using websites, podcasts, and online groups or forums. After each task, participants took part in an interview or completed an online survey to report and reflect on their experiences. Following this, 4 focus groups were run to gather a broader understanding of the role of technology in information seeking.

**Results:**

Websites afforded the greatest accessibility to digital menopause information, with participants reporting higher levels of competence and autonomy, primarily due to familiarity. Podcasts were the most novel of the 3 internet-enabled technologies for participants and also led to greater levels of competence and autonomy, particularly in comparison to the online groups or forums. Most participants found online groups or forums to be overwhelming and difficult to navigate. Across the 3 technologies, affective factors varied, markedly regarding levels of trust in information, but several key sources were relied upon. Engaging with internet-enabled technology for menopause information opened opportunities for participants to access peer experiences, which helped to normalize, validate, and understand their menopause experiences.

**Conclusions:**

It is important to consider how individuals engage with several different internet-enabled technologies for menopause information rather than investigating one at a time. This study highlights the nuances across websites, podcasts, and online groups or forums in terms of familiarity, accessibility, trust, and lived experience.

## Introduction

Menopause is characterized by the point in time at which a woman’s menstrual periods have stopped for 12 consecutive months [1]. This usually occurs naturally between 44 and 55 years of age but can be highly variable, and menopause can also be induced as a result of surgery, serious illness, or medication [2-4]. In an aging population, women spend one-third of their life perimenopausal, menopausal, or postmenopausal [5]. Perimenopause is the period before this point in time, and women often experience physical and physiological symptoms that can be confusing and affect their quality of life [6-8], including hot flashes, night sweats, weight gain, anxiety, and fatigue [9,10].

Despite the commonality of these experiences, many women report poor knowledge of the menopause transition and are less likely to seek or receive the medical assistance they require [7,8,11]. While menopause information can come from a variety of sources, such as friends, family, and health care professionals, women are increasingly turning to internet-enabled technologies for menopause information to increase their knowledge and understanding. Research has shown that women are higher users of online health information compared to men [12], reflecting a broader shift toward proactive and digitally mediated health engagement for women. This trend underscores the importance of digital health literacy, defined as the ability to seek, understand, and evaluate health information from electronic sources [13], and raises questions about accessibility and equity.

This is in line with rising expectations that individuals take ownership of their health [14], use their own judgment to seek medical advice, question health care professionals about their health [15,16], and increase their own knowledge and awareness [17]. The Health Belief Model suggests that well-informed individuals are more likely to participate in health care decisions and engage in health preventative and treatment behaviors [18]. This also aligns with the National Health Service’s (NHS’s) commitment to patient-centered, patient-led care, encouraging individuals to be active participants in their health management and decision-making [19]. Consequently, the internet is now often the first place people look for health information compared to friends, family, and health care professionals [20].

Since the COVID-19 pandemic, there has been a further surge in digital health resources to improve health communication. Specific to menopause, these resources, online information, and mHealth (mobile health) apps are often termed “Femtech” or “menotech” [21,22]. New technologies have changed the way we communicate; however, the abundance of menopause-related media content—ranging from podcasts and social media posts to online groups and message boards—is rarely governed by regulations [23]. While this content is often accessible and engaging, the quality and reliability can vary significantly, which may exacerbate inequalities in digital health literacy and access.

Existing evidence is perhaps limited, as noted in a recent literature review by Osborne and Sillence [24] that identified only 14 papers that considered the use of internet-enabled technologies for accessing menopause information. When engaging with these digital technologies for menopause information, women reported finding them accessible, convenient, and easy to use [22,25-28]. The main motivation for engaging with this content was centered on the desire for women to normalize, validate, and legitimize their own menopause experience as well as to understand their symptoms [22,26,29-31]. However, the extent to which someone trusted a source impacted whether an internet-enabled technology was accessed and how subsequent information on menopause was appraised [22,26,28,30-33].

A small body of research has found that general health information presented verbally increased health knowledge [34,35], more than written content [36], suggesting that audio-based materials may be an accessible and understandable means of communicating health information [25]. However, little research has explored how audio media can be specifically used for menopause or how they may serve dual roles in education and entertainment. Only a few papers have specifically considered the role of podcasts in relation to menopause [25,26], highlighting the importance of peer experiences in menopause information. Similarly, online group spaces and forums place peer experiences at the center, but to date, research that has considered this has focused on the data collection of posts and content rather than on women’s experiences of consuming the media content on menopause information in these settings [37-39]

Despite the pressure to be informed about one’s own health and the breadth of digital resources available on menopause, what remains poorly understood is how different internet-enabled technologies collectively shape women’s access to information. Existing research has largely examined individual technologies in isolation, overlooking the interplay between platforms and the contextual factors that influence engagement [24,40]. Examining women’s use of different technologies to access menopause information allows the exploration of commonalities in value and approach, as well as the potential to highlight challenges in digital engagement across the provision as a whole. It is important to consider the nuances between how the technologies are perceived and used to understand experiences and better inform future digital solutions regarding accessibility, representation, and sustainability of digital menopause information that facilitates meaningful and informative engagement.

One study that has attempted to examine women’s use of different digital technologies is that of Lupton and Maslen [41]. Although not focusing on menopause specifically, Lupton and Maslen [41] explored how Australian women use different types of digital technologies regularly for health-related purposes. Using a feminist new materialism perspective, they focused on the affordances, the associated affective factors, and the agential capacities created by these different technologies (eg, the ability to act confidently and make informed choices when engaging), including health information websites and online discussion forums. This approach to this study ensured rich findings into the nuances and complexity of technology use when engaging in online health information. This methodological perspective illuminates the pervasive nature of internet-enabled technologies for engaging with health information in the context of everyday life.

In this paper, we take a similar holistic approach to examine women’s engagement with websites, podcasts, and online forums as part of a digital ecosystem. Drawing from feminist new materialism and self-determination theory [42-44], we explore the affordances, affective factors, and the agential capacities of these technologies, alongside motivations and challenges related to autonomy (agency), competence (ability and effectiveness), and relatedness (connection). This dual theoretical lens enables a nuanced understanding of engagement and informs the design of inclusive, accessible, and trustworthy menopause resources.

This paper aims to explore how women engage with different internet-enabled technologies to access digital menopause information.

## Methods

### Design

**Figure 1.**
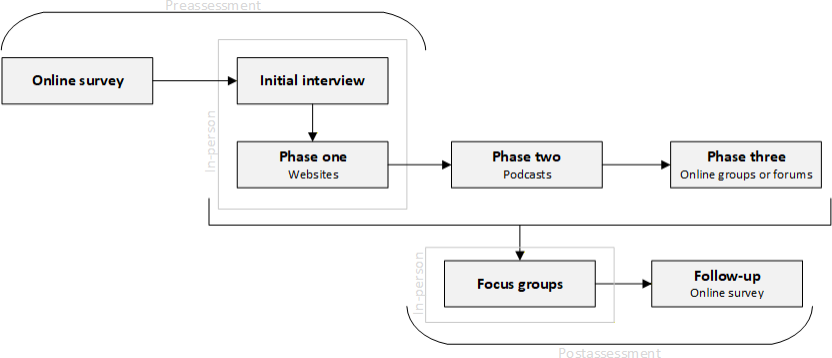
Study design overview.

A mixed methods, multiphase sequential design was used, split into 3 main parts: preassessment, primary data collection, and postassessment. Data collection was carried out using interviews, surveys, and focus groups at various stages throughout this study. See Figure 1 for an overview of this study’s design. This was an exploratory pilot study designed to generate rich, nuanced insights rather than generalizable findings. The focus was on depth of engagement across multiple digital technologies.

### Participant Recruitment

Women who identified as going through the menopause were recruited from the North East of England through purposive sampling (led by AKO), local organizations focusing on menopause and/or women’s health (eg, health centers or menopause cafes), university campus, and staff networks.

All participants took part in every stage of the research. This comprehensive participation ensured that detailed and nuanced experiences of accessing menopause information across an ecosystem of internet-enabled technologies were captured. Recruitment was ongoing in the early stages of the research and was stopped when data saturation was achieved during the preassessment interview. This saturation was achieved at each interview stage of this study.

### Preassessment

Participants initially completed a short 10-minute online survey to capture demographics, menopause experience, including their perceived knowledge of menopause, and self-reported digital engagement and ability. Following this, participants took part in an in-person 15-minute semistructured interview exploring the topics from the survey in greater detail (see Multimedia Appendix 1 for survey and interview schedule).

### Primary Data Collection

Participants engaged in 3 sequential phases of menopause information-seeking tasks using different internet-enabled technologies:

- Websites: 30 minutes searching for menopause-related information on websites using a desktop computer (in-person).
- Podcasts: listening to menopause-related podcast episodes for a minimum of 2 hours over 2 weeks using any personal device (at-home).
- Online groups or forums: engaging with menopause-focused online groups or forums for at least 1 hour over 1 week using any personal device (at-home).

Participants took part in an in-person 20-minute interview after the website’s task and completed an online survey after the podcasts and online groups or forums tasks, exploring participants’ experiences, motivations, challenges, perceived knowledge, and likelihood of future use through primarily textual responses.

Data on the sources of information that participants engaged with were also collected. To assess engagement beyond usability, the TENS-Interface (Technology-Based Experience of Need Satisfaction–Interface) [45] was administered after each task. This 15-item scale maps onto the 3 factors associated with self-determination theory, autonomy (agency), competence (ability and effectiveness), and relatedness (connection), to capture mastery, need satisfaction, and immersion (see Multimedia Appendix 1 for task sheets, interview schedule, and surveys).

### Postassessment

Participants attended 1 of 4 focus groups in-person (approximately 60 minutes) to reflect on their experiences across all technologies, considering accessibility, motivations, and complementary use. Further, 6 weeks later, participants completed a follow-up online survey to assess the lasting impact of engaging in these information-seeking tasks (see Multimedia Appendix 1 for focus group schedule and survey).

### Ethical Considerations

All participants were provided with an information sheet and asked to read it before completing a consent form, giving their full informed consent to take part. Following completion of this study, a debrief sheet was provided reiterating what this study was about, participants’ right to withdraw, and contact details for support if needed. All data from this study were anonymized. Participants received £60 (US $81.24) on completion of this whole study. This study was approved through Northumbria University’s ethical approval system (REF: 5289).

### Analysis

Audio files from interviews and focus groups were transcribed and collated with the qualitative survey data. Qualitative data were analyzed manually using visual and tactile techniques (eg, printed transcripts, annotation, Post-it [3M] notes, and paper-based thematic mapping) to support an iterative and collaborative analytic process. Participants’ quotes were physically extracted from transcripts and organized alongside codes on large-format thematic maps to aid the development and refinement of themes. Qualitative data were analyzed using reflexive thematic analysis [46,47] following a deductive, theory-informed approach grounded in feminist new materialism [41]. Data were analyzed for each technology individually before synthesizing cross-cutting themes with a focus on how participants described the affordances and affective factors of different internet-enabled technologies and how they impact the agential capacities of each of these. The 6-phase process outlined by the guided analysis of Braun and Clarke [46,47]: familiarization with the data, generation of initial codes, searching for themes, reviewing themes, defining and naming themes, and producing the report. In the final phase, 3 cross-cutting themes were identified that synthesized key patterns across all technologies and were guided by the theoretical lens. Coding was conducted collaboratively by AKO, ES, and CC, with iterative refinement and team review.

All quantitative data were entered into SPSS (IBM Corp) and analyzed. Descriptive statistics were collated to provide an overall understanding of participants’ digital skills, technology engagement, and TENS-Interface scores. ANOVAs were conducted on the TENS-Interface scores to assess statistical differences between the technologies.

Qualitative and quantitative data were collected across phases and analyzed separately, then integrated at the interpretation stage through triangulation. Quantitative findings informed cross-technology comparisons, while qualitative data provided contextual depth and explanatory insight into these patterns.

## Results

### Overview

The findings begin with an overview of participants’ demographics, then their experiences engaging with technology for menopause information, including their self-reported scores on competency, autonomy, and relatedness for each internet-enabled technology. The findings are then presented by internet-enabled technology—websites, podcasts, and online groups or forums—with a focus on the reported affordances, affective factors, and agential capacities of each. The Discussion section explores these technologies as part of a wider digital ecosystem, examining key similarities, differences, and emergent cross-cutting themes.

### Participant Demographics

A total of 16 women, aged between 40 and 62 (mean 49.9, SD 6.30) years, took part in every stage of this study. Most participants reported being at the perimenopause stage (n=10) and were currently taking hormone replacement therapy (HRT; n=9). For the participants, perimenopause began between the ages of 37 and 51 (mean 44.4, SD 4.40) years.

### Engaging With Internet-Enabled Technology

The initial interview and online survey explored participants’ experience with internet-enabled technologies for menopause information. All participants had experience of accessing menopause information online, describing their digital engagement as between “learning the ropes” and being “confident” in their abilities; all self-reported basic digital skills.

Participants reported which internet-enabled technologies they had used previously to access digital information on menopause and other health issues. Websites were the most commonly used to access menopause and other health information, followed by social media. More participants used podcasts, mobile apps, and social media for menopause information than for other health issues, whereas more had experience with websites for other health issues.

Beyond access to digital menopause information, 81% (n=13) of the participants had seen a health care professional at some point for menopause. The sources of menopause information accessed by participants in the information-seeking tasks were recorded throughout this study. The most commonly accessed resources were the UK NHS for websites, podcasts hosted by health care professionals, and charity-based online forums.

**Figure 2.**
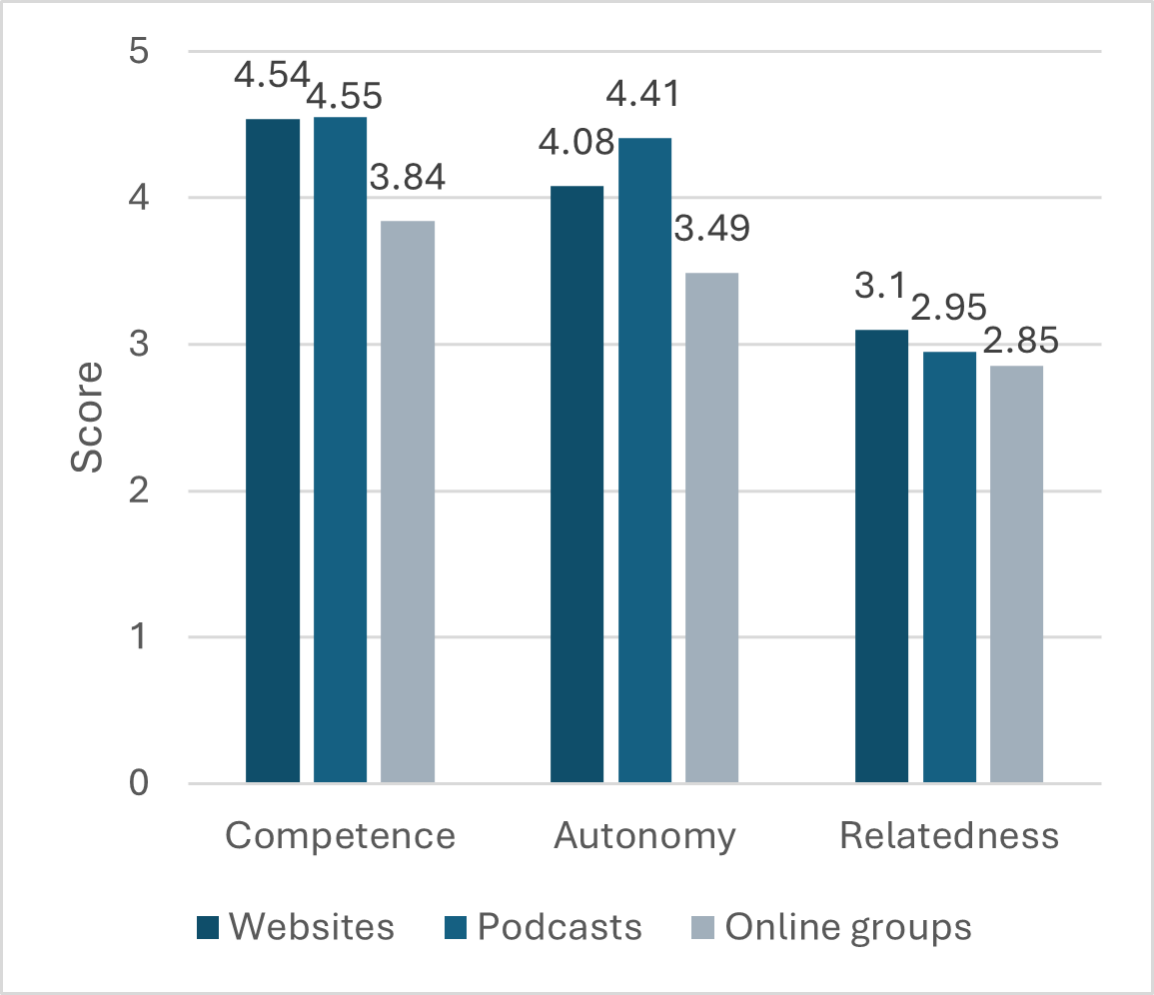
Competence, autonomy, and relatedness scores for websites, podcasts, and online groups, based on the TENS-Interface (n=16). TENS-Interface: Technology-Based Experience of Need Satisfaction–Interface.

After each information-seeking task, participants completed the TENS-Interface to explore their motivations to engage with the different technologies. This resulted in a score for competence, autonomy, and relatedness for websites, podcasts, and online groups (Figure 2). ANOVAs were then carried out with scores from all 16 participants.

There was a statistically significant difference between groups for competence, as determined by 1-way ANOVA (*F*_2,45_=6.883, *P*=.002). The effect size, eta squared (η²), was 0.234, indicating a large effect size. A Tukey post hoc test revealed competence scores were statistically higher for the websites (mean 4.54, SD 0.494, *P*=.007) and the podcasts (mean 4.55, SD 0.514, *P*=.006) compared to the online groups (mean 3.84, SD 0.807). There was no statistically significant difference between the scores for the websites and podcasts.

There was a statistically significant difference between groups for autonomy as determined by 1-way ANOVA (*F*_2,45_=7.816, *P*=.001). The effect size, eta squared (η²), was 0.258, indicating a large effect size. A Tukey post hoc test revealed autonomy scores were statistically higher for the websites (mean 4.08, SD 0.708, *P*=.04) and the podcasts (mean 4.41, SD 0.376, *P*<.001) compared to the online groups (mean 3.49, SD 0.839). There was no statistically significant difference between the scores for the websites and podcasts.

There were no statistically significant differences between groups for relatedness as determined by 1-way ANOVA (*F*_2,45_=0.461, *P*=.63). The effect size, eta squared (η²), was 0.020, indicating a small effect size.

### Menopause-Related Websites

In this study, menopause-related websites were defined as any website containing menopause information, ranging from single web pages to whole websites dedicated to menopause. These websites were very familiar to participants and were often the first resource used to find any health information, particularly for menopause. Participants described extensive experience using the internet, often daily, as part of their job or as a habit.

*It’s something I’m used to, it’s territory I’m familiar with, using the internet, browsing, you know, computer etc, so that was really familiar for me.*

[P14]

*I think it’s habit, and my habit has always been to just Google.*

[P13]

All participants used Google (Google LLC) as their search engine and found websites very accessible, easy to use, and had a lot of information on them.

*The internet, you’re straight in, you’re right, you just go straight to what you’re looking for, you find it.*

[P05]

Despite the participants’ ease of access, they emphasized the importance of approaching searches with a plan. Having a specific question was necessary to focus, manage the volume of information, and avoid becoming overwhelmed.

*Google is very clever. It is very, very clever, but sometimes it’s just what you put in. You’ve got to be very specific.*

[P07]

Descriptions of the risks of getting lost in “a rabbit hole” (P05 and P07) were common*,* highlighting the negative consequences of easily accessible digital information with heavily hyperlinked website designs.

Regarding design, participants discussed how different websites are from each other and that how they engage with them depends on the “success” of this design. Poorly designed websites often result in a negative experience. A couple of participants specifically explored the incongruence of the use of color to convey serious information, the connotations of certain colors, and issues around language:

*If you go onto a website that looks – that purports to be – talking about something serious, “Menopause, the truth,” but you go on and it’s all pink pastels, and flowers and dream catchers, instantly you're going to go, “That’s a load of bollocks,” and move away from it, for me.*

[P14]

*I find some of those websites have quite patronising language sometimes, which might make me sort of go hmm. It’s usually about weight gain.*

[P02]

Using a search engine such as Google left participants needing to make further decisions regarding which websites to access (tasks were carried out before the introduction of Google artificial intelligence in searches). Affective factors such as the familiarity of website names and the perceived trustworthiness of these, such as the NHS, were key in deciding which sites to visit.

*Everybody has heard of the NHS, and it’s really well trusted it just kind of made me want to visit first just to kind of see what they said first. Just for reputation really.*

[P09]

An “expert” or professional feel to the website also contributed to the perceived credibility, particularly in the case of the NHS. Trust extended to any websites that were linked from the NHS, as they were seen as “recommended” sources:

*If it is linked to the NHS website, you think, “oh, well, that must be a good resource to go to if they’re…” It’s almost like a recommendation isn’t it, that it’s there.*

[P04]

Participants valued a balance between professional expertise and personal experiences. The voice of the source was important, and P01 specified that it “always feels better when it has got a bit of a personal thing” on the websites. There was a dislike of commercialization on websites, advertisements, particularly linked to pharmaceutical companies or any organization that would make a profit, which led to distrust of the information available:

*I would steer clear of anybody who was asking me, I wouldn’t buy any product from a website… I just wouldn’t trust that at all.*

[P12]

*The top five or six [results] were ads for specific medications, it’s just like selling snake oil. It’s just like, “you know, this’ll change your life. You don’t need HRT,” so I avoided those kinds of things.*

[P02]

Most importantly, relevance was central to engagement. Participants sought information that best represented themselves, particularly UK-based content or the way in which menopause and women were portrayed. They expressed distrust of sources that were not aligned with UK health care or presented narrow representations of menopause.

*I normally put UK on the end because you often get a load of American stuff, which isn’t particularly relevant.*

[P04]

*We’re underrepresented, basically, because people think the menopause happens to people with long hair, husbands and children at home.*

[P12]

Engaging with menopause-related websites increased participants’ knowledge and confidence. Many reported that the information reinforced what they already knew about menopause; for others, they were able to validate their symptoms and experiences. This upskill in knowledge meant that participants felt that they were more prepared for what was to come, for those in the earlier stages of menopause, and could share their knowledge with others.

*Just thought “well I’ll read what they have to say about the things I possible already know.” They didn’t tell me anything new, in lots of ways, but they reinforced that the knowledge that I already have, basically, is okay.*

[P08]

*I was right to look, not before this session but over the last year… It’s good to have the foreknowledge, it’s not scary.*

[P05]

Accessing menopause information online also fostered a sense of shared experience and reduced feelings of isolation. This was evidenced by P02, who was unaware of the extent to which others shared their experience before engaging with technology:

*It’s been invaluable, a really good resource to look at what other people are saying about the menopause and realising you are not alone. Menopause with younger women is not common but it’s not wild, it’s not crazy.*

[P02]

### Menopause Podcasts

Menopause podcasts were defined as any podcast episode that focused on menopause. Almost all participants had little experience with podcasts and were new to the menopause-related content in this format. Engaging with podcasts was overwhelmingly positive, with participants often expressing surprise at how informative podcasts were:

*There is a lot that hasn’t crossed the mind regarding menopause related information and podcasts seem to be a great way to gain that, especially regarding current issues.*

[P13]

*I found the episodes I listened very engaging. I have not really considered listening podcasts on the topics previously and was positively supposed how informative the podcasts were.*

[P16]

Participants found podcasts to be very accessible, particularly the ability to listen while completing other activities such as household chores or driving. This also afforded participants the choice to be active or passive in engaging with the podcasts.

*Podcasts are the easiest to fit into your life because you can do it as you’re either driving or in the house pottering, so it’s an easy thing to fit in.*

[P03]

*Probably one of the things about podcasts as well is that you’re kind of allowing people’s expertise to filter the internet for you. And you can just sit back and be educated passively, you don’t have to do any of that searching.*

[P01]

However, some felt less engaged with audio information, often when the content was complex or required focused attention.

*There were a few that were too complicated to listen to whilst walking – felt like I needed a paper and pen and a few Google searches to follow the narrative.*

[P01]

For others, podcasts provided a sense of intimacy and personal connection when using headphones:

*I think the intimacy of a voice, if you’re wearing headphone in your ear or something there’s the confessional kind of element brought in and it does, yeah, it feels really intimate doesn’t it as opposed to just reading a story on the page.*

[P12]

Searching for specific menopause information within podcast platforms, whether through Spotify (Spotify Service), Apple Podcasts (Apple Inc), or a podcast-specific website, was challenging with search functions that were not intuitive. This difficulty sometimes led to unplanned engagement with broader or unexpected menopause-related topics.

*I was a bit overwhelmed by the lack of sorting or whatever algorithm Google does for me to make things easier to search for on Spotify and BBC. That was the main challenge.*

[P01]

*Some of the content I would not have initially looked to listen to, however this gave me a much wider understanding of the menopause across many different areas.*

[P07]

Regardless of the information participants accessed through the podcasts, decisions to continue listening were influenced primarily by the tone, style, and speaker credibility. A professional approach was favored with some personal experience or personal stories to “break up” the podcast and keep the format “light” and “conversational.” Medical expertise was considered essential for trust, even when personal narratives were present.

*What I preferred, I loved the funny remarks within some of the episodes, they made me smile, whereas soe seemed to concentrate on the negative aspects of the menopause.*

[P15]

*It’s a conversation, rather than you’ve gone to a presentation or something like that, it’s more a conversation.*

[P01]

*I though the content of them was good, liked that they had medical professionals providing information and not a self-appointed expert… I just wanted someone who definitely knew the science behind the process properly.*

[P06]

As with websites, participants favored podcasts that covered topics that were relevant to the participant, especially those with a national or local application. There was a lack of interest or engagement with US-based content, often due to commercial undertones and the differences in health care between the 2 countries.

*I was less engaged with the US podcast so probably would avoid that particular one in the future.*

[P04]

Participants reported they were able to digest tangible information and share this with friends. Several affective factors were linked with the usage of the podcasts, specifically not feeling alone. This was most prominent in reflections centered on the practice of sharing peer experiences of menopause, which helped to normalize menopause.

*Women are not alone and what we are going through is normal.*

[P09]

*Until I listened to that podcast, I was generally worried. I thought, “my God, what’s happening?”*

[P10]

Overall, the menopause podcasts left participants feeling “less alone and more empowered to talk about the menopause with other people” (P01) and to be proactive in their own health, self-monitoring potential changes.

*I thought I was well informed and surprise I am not, so they have made me feel more empowered to do something about my menopause.*

[P06]

### Online Groups and Forums for Menopause

Participants encountered several online groups and forums dedicated to menopause. Experiences varied depending on their familiarity with platform design. P14, a Facebook (Meta) user, found the groups on this platform to be “easy to engage.” However, the most common response to accessibility and navigation of online groups or forums was more negative. There was a lot of content available, and navigation was frequently described as difficult and overwhelming, leading to difficulties in processing the information and going “down menopause rabbit holes” (P05) for extended periods of time.

*Just being overwhelmed by the sheer tonne of information and stream of people’s personal horror stories that flood your brain.*

[P12]

*There was a wealth of info to read and digest and I spent so much time on that I didn’t actually progress to anything more involved.*

[P04]

The volume of information available often led to extended time spent reading, with participants reporting difficulty identifying relevant or trustworthy information. Many felt that engagement was time-consuming and offered limited educational benefit. P02 reflected on “loads of conflicting information” that was “difficult to sift through.”

*It was awful, such a nightmare to try and negotiate. Like I said, I am not savvy on them, so I may not have been used to the way to find things… it’s so time consuming.*

[P15]

*I found it took a while to sift through information and do an awful lot of reading which is sometimes challenging after being at work on a computer all day.*

[P02]

Online groups or forums afforded the participating women with numerous ways to interact with posts, whether this be through liking, sharing, or commenting. This is different compared to general websites or even podcasts. Many participants could see a benefit of this approach for certain individuals, but did not feel comfortable engaging or interacting in this way themselves. This was often due to the public nature of the forums, reporting they “didn’t feel “brave” enough to ask or answer any questions” (P05). Overall, the group conveyed a preference to only read the posts.

*I am very wary of the risks of engaging online… I have never commented online, I am a “lurker” – I read only. I feel reading is enough for me, I don’t need more engagement as yet.*

[P12]

Primarily, the posts that participants encountered were of women sharing their experiences of menopause, whether this be to ask questions, give tips, or to “vent.” The participants valued these stories; they were “comforted by the stories in the community” (P12), they seemed “real and the answers heartfelt” (P05). There was a focus on “the humanity of it, the stories” (P12). Reading about peer experiences of menopause provided a measure of connection, which ultimately made participants feel as though they were not alone in their own menopause journey and that there was a supportive community.

This was particularly evident for participant P12, who reflected on how online groups or forums could be “good for calming you down when you feel you are the only one suffering from a particular symptom,” as it can be “very reassuring to read others’ experiences and it usually introduces you to some new medication/approach which you can follow up.” Participants did not always feel that the online groups or forums worked for them, but reflected a lot on the benefits for others. It was important to them that any information gained through this mode was considered in the context of information gathered through other sources. The very personal nature of the content on the posts sometimes contradicted their own experience or knowledge of menopause.

*In the groups, people are putting their individual experience on, so maybe contradicts with what you thought you had learned.*

[P01]

Concerns were raised about the credibility of information shared in forums, particularly due to the absence of identifiable medical expertise and the anonymity of contributors.

*You don’t know how you’re talking to as well. You trust the podcast because you know it’s Doctor Newson, or whoever’s online, you could be talking to Bill who’s 59.*

[P10]

*It just felt like everyone was almost looking for help and trying to find an answer, but nobody there had the answer. There wasn’t a doctor there or a medical professional or an expert or someone, even your Davina’s of the world.*

[P14]

The large volume of information was overwhelming; P15 explained that “the challenge is getting to the source of what you want.” Participants described feeling less confident in their knowledge after engaging with online groups or forums, sometimes doubting previously trusted information. Emotional responses were also notable, with many describing feelings of sadness or distress when reading others’ experiences.

*This was one thing I picked up from Mumsnet was there was so much stuff on there that you think, “well perhaps I don’t know as much as I thought I knew.”*

[P04]

*There was almost quite despairing, quite sad feel to them if I put it in those terms, whereas the rest of the information I felt in control of.*

[P14]

Participants voiced a lack of control regarding seeing this information, and some felt it was important to use strategies to deal with encountering negative posts and posts that were not relevant. They also expressed wanting to stop the experience from becoming overwhelming for them.

## Discussion

### Principal Results

This study explored how women engage with different internet-enabled technologies to access and consume digital menopause information. Unlike previous research that has focused on single technologies in isolation, this study adopted a holistic approach, examining websites, podcasts, and online groups or forums as part of a wider digital ecosystem. With the exception of the study by Lupton and Maslen [41], few studies have taken this comparative approach. Drawing on this paper, feminist new materialism and self-determination theory, this study examined how the affordances, affective factors, and agential capacities of different technologies shaped engagement, alongside motivations and challenges relating to autonomy, competence, and relatedness.

A total of 16 women experiencing menopause participated across all phases of this study, providing rich comparative insights. The exploratory design allowed for depth and detail. Each internet-enabled technology offered distinct affordances, affective factors, and agential capacities, shaped by participants’ familiarity, digital skills, and preferences for accessing and consuming health information. Websites and podcasts were associated with higher competence and autonomy scores than online groups or forums, suggesting these technologies may better support self-directed and confident information-seeking. Relatedness scores did not differ significantly across technologies, likely reflecting the general nature of this study’s tasks rather than a focus on support-seeking.

Overall, the findings highlight how different internet-enabled technologies shape menopause information-seeking in nuanced ways and point to important implications for the design of inclusive, trustworthy digital health resources. Discussed below, this work begins to develop our understanding of the interplay between different internet-enabled technologies, the format of media content, and the consumer’s personal and contextual preferences.

### Familiar, Accessible, and Navigable Technology: Overcoming Information Overload

Across internet-enabled technologies, participants discussed the overwhelming nature of menopause information, frequently referencing going “down a rabbit hole.” This is unsurprising as internet users are often presented with a large amount of information, which is often irrelevant [48]. However, in terms of menopause information, this contradicts existing research suggesting there is a paucity of readily available, relevant, reliable, and accessible resources of menopause information [27].

Issues arise when searchers are unable to effectively filter and evaluate content, leading to information overload that can negatively affect engagement and information processing [49]. This is particularly prominent with health-related media, and research has acknowledged that this has become a widespread problem in patients and physicians [49,50]. Zhong et al [51] further demonstrated the effect of information overload on mHealth users’ perceived symptom severity, susceptibility, treatment benefits, barriers, self-efficacy, and action cues.

In this study, participants appeared better able to manage overload when using websites and podcasts. Familiarity with search engines and prior digital experience enabled participants to navigate information effectively, illustrating the role of digital health literacy in mitigating overload. Websites, in particular, were the most familiar due to the frequency of use in their everyday lives and were perceived as accessible, contributing to higher autonomy and competence scores. This aligns with evidence that the internet is often a first source for health information [20,52] and can support empowerment and informed decision-making [53]. Specific to menopause information, research has indicated that women have the greatest experience with health websites and the lowest experience and awareness of digital health apps [29], but that technology as a tool to access menopause information is accessible, convenient, and easy to use [24].

The way in which a technology is seen as accessible can be different for each person. In comparison to the familiarity participants had with websites, podcasts were the most novel technology. Only 21% of participants had any experience of using podcasts for menopause information, mirroring findings in the study by Harper et al [54], where 20% had used podcasts. Podcasts afforded participants the option to engage with menopause content actively or passively, while carrying out other tasks such as driving or housework. This was a key motivation for engaging with podcasts, as well as the convenience and accessibility. Few research papers have considered the role of podcasts in menopause information, but these findings support qualitative work by Shaw et al [26] and broader literature highlighting the portability as a strength of audio media (eg, the study by Weiner [55]).

In contrast, navigability was more problematic for podcasts and especially online groups or forums. Participants valued Google’s ability to filter and prioritize relevant information—an affordance not replicated in podcast platforms or forums. While competence scores for podcasts remained high, online groups or forums elicited significantly lower autonomy and competence scores. Information overload in these spaces reduced participants’ sense of control, trust, and perceived knowledge, consistent with research showing that overload can lead to disengagement and negative attitudes toward digital technologies [56]. This was evidenced in participants’ reflections on future engagement with this technology, their lack of trust in the authenticity of the content, and their feeling less knowledgeable about menopause. In this case, the information overload compromised participants’ perceived control over searching for relevant and reliable content more than improving it [57] and was reflected in the autonomy scores. In comparison to websites and podcasts, online groups or forums reported significantly lower scores on autonomy and competence, and reported feeling less knowledgeable about menopause after engaging with the platform.

### Assessing the Trustworthiness of Menopause Information Sources

Motivations for engaging with the different internet-enabled technologies were shaped not only by functional affordances but also by affective factors, particularly perceived trustworthiness. This trust extended beyond the information source to the technology itself as a mediator of content. Participants favored familiar and recognized sources, particularly evidence-based, medical providers. For websites, trust was strongly associated with reputable organizations such as the UK NHS website. Participants relied on established visual and institutional cues to assess credibility, and issues arose when this was incongruent. For example, the use of certain colors, such as pink, to convey serious health information, and the connotations of these colors. As noted in the literature review by Osborne and Sillence [24], menopause information is most trusted when sourced from medical or expert organizations [28,33]. This reliance upon major sources of health content, such as hospitals, national health services, and health care professional websites for trustworthy information, is not specific to menopause and has been evidenced in wider women’s health research [41,58]. Importantly, participants appeared to trust websites as a technology before evaluating individual sources, suggesting a preexisting confidence in this technology.

Emphasis on trust was slightly different for podcasts. While familiarity with presenters (eg, celebrities or influencers) remained important, participants also considered topic relevance and popularity. The podcasts facilitated a sense of intimacy, fostering a connection with the presenters and stories, a characteristic feature extensively documented in podcast research [59-62]. Similarly, Shaw et al [26] noted that relatability and storytelling were central to engagement with menopause podcasts. Podcast listeners may also form stronger parasocial relationships with the presenters when they share interests and personal experiences [63].

The development of this trust in podcasts was a direct contrast to the difficulties participants experienced with establishing trust in online groups or forums. Concerns centered on anonymity, authenticity, and lack of visible expertise. Considering the engagement framework from Sillence et al [64], these findings suggest that without baseline trust in the platform, engagement and positive outcomes are compromised. It is not just the media content source that participants did not find credible, but the affordances of the internet-enabled technology itself—the poor navigation and prior reputation of online groups or forums.

Despite the qualitative data in this study emphasizing the value of lived experiences, relatedness scores across the 3 technologies were low. This may reflect the general nature of the tasks, which focused on information-seeking rather than explicit support. Participants’ varied interests and motivations likely contributed to this pattern.

However, regardless of which internet-enabled technology, participants expressed strong mistrust of commercialized content. This has been a consistent finding across the literature for digital menopause information, where any advice and information linked to pharmaceutical companies or for-profit organizations was mistrusted [26,28,33].

### Access to Lived Experience Menopause Stories

Lived experience narratives played an important role in shaping trust and engagement, particularly within podcasts and online groups or forums. Podcasts were favored for the “humanness” of hearing women’s stories, making them easier to connect to, supporting previous work highlighting the role of narratives in menopause communication [26,28].

Engaging with lived experiences enhanced agential capacities by reducing feelings of isolation and increasing empowerment to discuss menopause openly. To some extent, these stories became a source of validation, allowing participants to reframe their own menopause experiences. This was less evident for websites, where information-seeking focused primarily on trusted, medical content and sources (eg, NHS). These findings align with those of Osborne and Sillence [24], who note that motivations for engaging with digital menopause resources often include a desire to validate and legitimize menopause experiences through diverse stories [22,25,26,31]. Peer experiences not only promote normalization but also frame menopause in a relatable way, fostering a sense of community and reassurance that others are navigating similar challenges [22,25].

Despite this, participants in this study predominantly reported negative experiences with online groups or forums. These internet-enabled platforms elicited several negative affective factors, with participants reporting dissatisfaction with the format, emotional overload, and distrust in the credibility of content. This contrasts with existing menopause research suggesting online peer support can be supportive and reduce feelings of loneliness [22,27]. A prior literature review by Eysenbach et al [65] might provide some insight into these mixed results, suggesting the benefits of online communities depend on an individual’s intrinsic motivation to engage socially. Participants in the current study often acknowledged the potential benefits of online groups or forums for others but did not feel such spaces suited their own needs.

Recent research by Dallolio et al [66] further supports this nuance, illustrating that while online groups or forums can provide valuable support and facilitate emotional exchanges, particularly for women who lack proper health care and support during menopause, they may not be universally beneficial. Importantly, podcasts were perceived as more reliable sources of lived experience because narratives were typically curated and facilitated by experts, enhancing their credibility and coherence. This professional yet personal approach aligns with earlier work on online health communication, where trust and relevance are key factors in determining the success of health-related media content [67]. Sillence et al [68] found that while people value the authenticity of peer experiences, they often question their reliability [68,69]. This suggests that professional oversight or facilitation might mitigate concerns over the credibility of shared information.

The tone and ethos of an online community were also critical. In this study, participants described some of these groups as platforms primarily used for venting about menopause experiences. While some participants found therapeutic value in these exchanges, others felt they were overwhelming or unhelpful. The value of these communities depends on individual preferences—some may seek emotional support and validation, while others may prefer more structured or evidence-based discussions. Armstrong et al [70] emphasize the importance of community identity and tone in online health spaces. When the tone of a community does not align with an individual’s needs—such as focusing on venting or specific treatments (eg, HRT)—it can detract from the perceived usefulness of the stories shared, leading to disengagement. This misalignment may explain the negative affective factors reported by some participants in this study. Consequently, the efficacy of online peer communities in menopause health communication may be influenced by how well the platform matches the users’ expectations and emotional needs.

Ultimately, the evidence suggests that the format and credibility of menopause-related digital content, particularly when it includes both professional guidance and lived experience narratives, play significant roles in shaping engagement and trust.

### Limitations

All participants self-reported good digital literacy skills and familiarity with digital technology for health information-seeking. As a result, findings may not reflect the experiences of individuals facing challenges with usability, navigation, or accessibility. Future research should explore these barriers, considering factors such as technical access, autonomy of use, social support networks, and experience [71]. Building on the in-depth insights provided here, there is clear value in scaling this work to a larger survey. A broader, quantitative study could examine patterns of access and engagement across websites, podcasts, and online forums within a more diverse population, complementing the nuanced findings presented here and offering a more comprehensive understanding of digital health behaviors in the menopause context. Additionally, not enough detail is understood regarding the potential role of online groups or forums. It is possible that the conflicting information in this study is a result of the nature of the tasks and the focus on upskilling participants’ menopause knowledge rather than a more specific purpose. Research with a greater emphasis on more organic information searching methods may help to mitigate this limitation.

### Conclusions

Different internet-enabled technologies play a distinct role in accessing menopause information. Websites remain the most familiar and trusted internet-enabled technology, supporting effective evaluation of sources and navigation of information. Podcasts, though novel for many participants, were highly valued for their accessibility, intimacy, and facilitation of content by professionals. Trusting in the source of menopause content was key in the motivations to engage with a technology and use menopause information, presenting challenges for online groups or forums where participants reported distrust, navigational difficulties, and emotional overload. While online forums may offer benefits for women lacking support networks, they were not universally perceived as helpful.

Across all internet-enabled technologies, lived experience stories were valued, though engagement depended on personal preferences, experience, trust, and availability of wider support networks. By examining multiple technologies together, this study provides a nuanced understanding of how format, credibility, and lived experience shape engagement. These insights inform the design of inclusive, trustworthy menopause resources and highlight opportunities to scale this work to larger studies for broader population-level insights.

## Supplementary material

10.2196/78215 Multimedia Appendix 1 Data collection materials.

## Abbreviations

HRT: hormone replacement therapy
mHealth: mobile health
NHS: National Health Service
TENS-Interface: Technology-Based Experience of Need Satisfaction–Interface
