# Supplementary material for: Women’s Engagement With Different Internet-Enabled Technologies to Access Digital Menopause Information: Mixed Methods, Multiphase Sequential Study
Source: J Med Internet Res. 2026 May 20;28:e78215. doi: 10.2196/78215 (PMC13189256; doi:10.2196/78215)
Supplement: Multimedia Appendix 1 — Data collection materials. [file jmir-v28-e78215-s001.docx]

**INITIAL SURVEY**

**Information sheet..**

**Consent form..**

**Demographics**

Please provide a four-character code or sequence as your unique participant code. We suggest your initials and year of birth (e.g., FL70).

*You will be asked for this code multiple times throughout the study in order to link your data, please keep a note of this. If you wish to withdraw your information from this study, please quote this code. This will allow us to remove your data and ensure your confidentiality.*

[open answer]

What is your age?

*Please enter your age in number of years.*

[open answer]

What gender do you identify with?

Woman, Man, Non-Binary, Prefer not to disclose, Self-Identify (describe)

Do you identify as transgender or have a transgender history?

No, Yes, Prefer not to disclose

**Menopause Experience**

What point of the menopause transition best describes your current experience?

Perimenopause, Menopause, Post Menopause

How knowledgeable do you feel you are about menopause?

No Knowledge, Little Knowledge, Some Knowledge, Knowledgeable, Very Knowledgeable *[Likert Scale 1-5]*

Have you ever looked for digital information on menopause? This could be using the internet, or social media or podcasts.

Yes, No

Have you had any communication with a Health Care Professional (e.g., a GP) about your menopause experience or symptoms?

Yes, No

Do you have any history of Hormone Replacement Therapy to treat any symptoms of menopause?

Yes, I am currently taking HRT, Yes, I have previously taken HRT, but I am not currently, No, I have never nor am I currently taking HRT

**Digital Skills**

Which of the following would best describe your engagement with digital technology?

*This could include anything from using the internet to accessing apps or using podcasts.*

1. *Never have, never will*
2. *Was online, but no longer*
3. *Willing and unable*
4. *Reluctantly online*
5. *Learning the ropes*
6. *Task specific*
7. *Basic digital skills*
8. *Confident*
9. *Expert*

*[Scored 1-9]*

Please score yourself on how comfortable/able you are doing the following tasks online.

|  | Can't do/don't know what it is | Would need help to do | Could do with difficulty | Could do | Expert (could teach others) |
| --- | --- | --- | --- | --- | --- |
| Send an email |  |  |  |  |  |
| Delete spam emails |  |  |  |  |  |
| Find stuff using a search engine such as Google |  |  |  |  |  |
| Watch a video on YouTube or iPlayer |  |  |  |  |  |
| Fill out an application form or buy something online |  |  |  |  |  |
| Use a mobile app |  |  |  |  |  |
| Evaluate whether a website is safe/can be trusted |  |  |  |  |  |

*[Scored - 0 = Can't do/don't know what it is, 1 = Would need help to do, 2 = Could do with difficulty, 3 = Could do, 4 = Expert (could teach others)]*

**Contact**

**We wish to invite you to campus for the next part of the study.**

This will be a short interview to introduce you to a member of the research team, and further explore your answers to this survey you have just completed. Following the interview, we will ask you to complete a task – searching the internet for information on menopause – with a discussion afterwards regarding your experience.

Could you please confirm the email address that would be best to contact you with to arrange a suitable date and time to come to campus for the next part of the study? *

[open answer]

** answer is required for this question.*

**------End of Survey------**

[contact information]

**INITIAL INTERVIEW SCHEDULE**

Thank participant for coming onto campus for this part of the study.

Confirm they have read the participant information and ask if they have any questions.

Go through the study plan with the participant.

We just wanted to take this time to consider your answers to the survey you completed in more detail to get a better understanding of what you have done to this point in gathering information on menopause.

You noted in your survey that you would describe yourself to be [perimenopause / menopause / post menopause], could you please explain what this means to you?

How knowledgeable do you feel about menopause?

*Prompt: menopause in general as well as specific to their own symptoms/experiences.*

How did you come to this knowledge on menopause?

*Prompt: could be through friends and family, online, HCPs.*

Have you ever needed to look for more information on menopause? If so, where do you go to find this?

*Prompt: family, friends, HCPs, digital information.*

Explore this experience further

If digital information is not mentioned before…

What experience, if any, do you have of searching for digital health information?

*Prompt: general health information and menopause specific.*

What types of technology did you use to access this information? (e.g., internet, social media, online forums, podcasts, mobile apps)

How did you find this experience?

How comfortable were/are you using technology to search for health information?

NOW INTRODUCE PHASE ONE TASK – INTERNET SEARCH

**INTERNET TASK**

**Internet Search for Information on Menopause Task**

Thank you for taking part in this study.

You will be given 30 minutes to search the internet for information on menopause. We ask that you complete this task however you would normally begin searching for online health information.

If you are struggling with a place to start, we recommend starting with a search engine such as Google with terms such as *‘menopause information’* or *‘menopause treatment’*. Alternatively, we have included a few example websites:

<https://www.nhs.uk>

<https://healthtalk.org>

<https://www.menopausematters.co.uk>

<https://www.womens-health-concern.org>

<https://www.daisynetwork.org/>

*Please note these are just suggestions and are not meant as required listening list or an endorsement of the content.*

The sound is on and headphones are available if there is any audio you wish to listen to.

**INTERNET SEARCHES INTERVIEW SCHEDULE**

How did you find the task?

How did you get started?

Did you have a question in mind that you were looking to find the answer for?

How did you find the experience of searching for information on menopause this way?

Thinking about the content, accessibility,

Refer to recording and highlight a number of websites to prompt conversation. Focus on:

Types of websites accessed – e.g., NHS/healthcare, pharma, community-based

Motivations for choosing websites

Why did they stay/leave the website(s)

Were there any websites they avoided altogether and why was this

What will you take away from this exercise today?

Prompt: search more, increased knowledge, speak to others, change in personal behaviour towards menopause experience… goal-directed outcomes vs. introspective outcomes

Was there anything about being recorded in this scenario that made you not look at something online or carry out this task differently? Explain….

If at home, would you have looked at anything differently? – How and why…

Would you search the internet again for information on menopause or for other health information?

**QUANT QUESTIONS**

To what extent do you agree or disagree with the following statements?

|  | Strongly Disagree | Disagree | Neither Agree or Disagree | Agree | Strongly Agree |
| --- | --- | --- | --- | --- | --- |
| I think that I would like to use the internet frequently. |  |  |  |  |  |
| I found the internet unnecessarily complex. |  |  |  |  |  |
| I thought the internet was easy to use. |  |  |  |  |  |
| I think that I would need the support of a technical person to be able to use the internet. |  |  |  |  |  |
| I found the various functions on the internet were well integrated. |  |  |  |  |  |
| I thought there was too much inconsistency on the internet. |  |  |  |  |  |
| I would imagine that most people would learn to use the internet very quickly. |  |  |  |  |  |
| I found the internet very cumbersome to use. |  |  |  |  |  |
| I felt very confident using the internet. |  |  |  |  |  |
| I needed to learn a lot of things before I could get going with the internet. |  |  |  |  |  |

[Scored – 1 = Strongly Disagree, 2 = Disagree, 3 = Neither Agree or Disagree, 4 = Agree, 5 = Strongly Agree]

Having carried out searches on the internet, how knowledgeable do you feel you are about menopause?

No Knowledge, Little Knowledge, Some Knowledge, Knowledgeable, Very Knowledgeable [Likert Scale 1-5]

How much do you agree with the following statement: “I learned something new about the menopause from searching the internet”

Strongly Disagree, Disagree, Neither Agree nor Disagree, Agree, Strongly Agree

[Likert Scale 1-5]

Would you gather information on menopause using the internet again?

Yes, No

Would you recommend podcasts to others for health information?

Yes, No

Reflecting on your experience searching the internet for menopause information, please rate your agreement with the following statements:

*Likert scale: 1 (Do Not Agree) – 5 (Strongly Agree)*

1. I feel very capable and effective at using the internet.
2. I feel confident in my ability to use the internet.
3. Learning how to use the internet was difficult.
4. I found the interface and controls confusing.
5. It wasn’t easy to use the internet.
6. The internet provides me with useful options and choices
7. I can get the internet to do the things I want it to.
8. I feel pressured by the internet.
9. The internet feels intrusive
10. The internet feels controlling.
11. The internet helps me to form or sustain relationships that are fulfilling.
12. The internet helps me to feel part of a larger community.
13. The internet makes me feel connected to other people.
14. I don’t feel close to other users of the internet.
15. The internet doesn’t support meaningful connections to others.

**PODCASTS TASK**

**Listening to Podcasts for Information on Menopause Task**

Welcome to the second task, thank you for your continued support of this study.

Over the next two weeks, we would like you to listen to *at least* four 30-minute podcast episodes on the topic of menopause - episode timings may vary but a minimum total of two hours. The episodes may cover anything as long as they are menopause related.

We ask that you complete this task however you would normally access/listen to podcasts. However, if you are new to podcasts or are struggling with a place to start, you can access podcasts in a variety of places including: online or through Spotify, Google Podcasts, Apple Podcasts, BBC Sounds, or Amazon Music. We have also included a number of free podcasts below as examples to get you started:

The Happy Menopause – [Google Podcasts](https://podcasts.google.com/feed/aHR0cHM6Ly9mZWVkcy5idXp6c3Byb3V0LmNvbS8zNjQ5NjQucnNz?sa=X&ved=0CAMQ4aUDahcKEwjAsquXq6CCAxUAAAAAHQAAAAAQNg)

The Latte Lounge Podcast - [Spotify](https://open.spotify.com/show/3vSZllHwOLlZkMISM0uEI8), [Online](https://www.lattelounge.co.uk/the-latte-lounge-podcast/), [Apple Podcasts](https://podcasts.apple.com/gb/podcast/the-latte-lounge-podcast/id1626848207)

The Dr Louise Newson Podcast – [Spotify](https://open.spotify.com/show/7dCctfyI9bODGDaFnjfKhg), [Google Podcasts](https://podcasts.google.com/feed/aHR0cHM6Ly9mZWVkLnBvZGJlYW4uY29tL1RoZURyTG91aXNlTmV3c29uUG9kY2FzdC9mZWVkLnhtbA?sa=X&ved=0CBsQ27cFahcKEwjAsquXq6CCAxUAAAAAHQAAAAAQLA), [Amazon Music](https://music.amazon.co.uk/podcasts/975ceea6-4c49-4c8d-b528-af85c5601668/the-dr-louise-newson-podcast)

Menopause Matters – [Apple Podcasts](https://podcasts.apple.com/gb/podcast/menopause-matters/id1689527177), [Spotify](https://open.spotify.com/show/3utaFq0WCuBbgVb7LZXcnL)

Menopause: Unmuted – [Online](https://www.menopauseunmuted.com/), [Spotify](https://open.spotify.com/show/6L5PwH8dmh7SrhMgfTBjpz)

Thriving in Menopause – [Spotify](https://open.spotify.com/show/6ukEKFBocz2TDn5Ev8ZtJr), [Online](https://www.preventionaus.com.au/thriving-in-menopause)

*Please note these are just suggestions and are not meant as required listening list or an endorsement of the content.*

As you listen to the podcast episodes, could you please keep a log of which you have listened to and which platform you access them on. To support this, we also ask that you complete this [Google Form](https://forms.gle/jfjkrocLSUWosMHm6) after each episode.

All podcast episodes you listen to will remain confidential.

At the end of the two weeks, a member of the research team will be in touch via email to ask you to complete an online survey regarding your experience of this task.

**PODCASTS TASK SURVEY**

**Welcome back!**

Thank you for your continued support for this study.

We hope you have had a chance to listen to at least four 30-minute podcast episodes on menopause. The following pages will ask you to reflect on this experience.

If you have any problems, please get in touch with a member of the research team:

[emails]

What is your participant code?*

*This is the four-character code you were asked to create at the beginning of the study. We suggested your initials and year of birth (e.g., FL70).*

[open answer]

** answer is required for this question.*

Over the last two weeks, how many podcast episodes did you listen to?

[open answer]

Could you please explain which podcast episodes you listened to and why.

*Where possible, please also indicate where you listened to the podcasts, e.g., Spotify, BBC Sounds, Apple Podcasts.*

[open answer]

Were there any podcast episodes that you considered but decided against listening to?

No, Yes

(Selecting yes…) *Please describe your reasons for not listening.*

Which of the following best describes how you listened to the podcast episodes?

Back-to-back, Multiple in one day, Spread across one week, Spread over two weeks, Other (describe)

If possible, could you please explain why you listened to the podcast episodes in this timeframe.

[open answer]

What was your experience of listening to podcasts to gain information on menopause?

*Consider how you engaged with the episodes, how they made you feel, whether there were any differences between the episodes...*

[open answer]

What do you think about the content of the podcast episodes?

[open answer]

What was good and bad about the experience?

[open answer]

To what extent do you agree or disagree with the following statements?

|  | Strongly Disagree | Disagree | Neither Agree or Disagree | Agree | Strongly Agree |
| --- | --- | --- | --- | --- | --- |
| I think that I would like to use podcasts frequently. |  |  |  |  |  |
| I found the podcasts unnecessarily complex. |  |  |  |  |  |
| I thought the podcasts were easy to use. |  |  |  |  |  |
| I think that I would need the support of a technical person to be able to use podcasts. |  |  |  |  |  |
| I found the various functions in the podcasts were well integrated. |  |  |  |  |  |
| I thought there was too much inconsistency in the podcasts. |  |  |  |  |  |
| I would imagine that most people would learn to use podcasts very quickly. |  |  |  |  |  |
| I found the podcasts very cumbersome to use. |  |  |  |  |  |
| I felt very confident using podcasts. |  |  |  |  |  |
| I needed to learn a lot of things before I could get going with the podcasts. |  |  |  |  |  |

[Scored – 1 = Strongly Disagree, 2 = Disagree, 3 = Neither Agree or Disagree, 4 = Agree, 5 = Strongly Agree]

Thinking about accessibility and the technical aspects of podcasts, how did you find engaging with the podcasts?

[open answer]

Having listened to the podcasts, how knowledgeable do you feel you are about menopause?

No Knowledge, Little Knowledge, Some Knowledge, Knowledgeable, Very Knowledgeable [Likert Scale 1-5]

How much do you agree with the following statement: “I learned something new about the menopause from listening to the podcasts”

Strongly Disagree, Disagree, Neither Agree nor Disagree, Agree, Strongly Agree

[Likert Scale 1-5]

Would you gather information on menopause using podcasts again?

Yes, No

Could you please explain your answer?

[open answer]

Would you recommend podcasts to others for health information?

Yes, No

Could you please explain your answer?

[open answer]

What will you take away from the Podcasts task?

[open answer]

Reflect on your experience using podcasts and rate your agreement with the following statements:

*Likert scale: 1 (Do Not Agree) – 5 (Strongly Agree)*

1. I feel very capable and effective at using podcasts.
2. I feel confident in my ability to use podcasts.
3. Learning how to use podcasts was difficult.
4. I found the interface and controls confusing.
5. It wasn’t easy to use the podcasts.
6. The podcasts provided me with useful options and choices.
7. I can get the podcasts to do the things I want it to.
8. I feel pressured by podcasts.
9. Podcasts feels intrusive.
10. Podcasts feels controlling.
11. Podcasts help me to form or sustain relationships that are fulfilling.
12. Podcasts help me to feel part of a larger community.
13. Podcasts make me feel connected to other people.
14. I don’t feel close to other users of podcasts.
15. Podcasts don’t support meaningful connections to others.

**------End of Survey------**

[contact information]

**ONLINE GROUPS / FORUMS TASK**

**Engaging with Peers Online for Information on Menopause Task**

Welcome to the third task, thank you for your continued support of this study.

Over the next week, we would like you to engage with online groups with a focus on menopause for at least one hour. We ask that you engage with any online groups of your choice and in a manner that comes most natural to you. For example, asking/posting questions, answering posts and /or simply reading posts and comments.

What do we mean by online groups? Any group or community online where you can read peer experiences of menopause. This may include, but is not limited to forums, message boards, Facebook Groups, Twitter/X Communities

If you are new to online groups or are struggling with a place to start, you can consider the following online forums as examples to get you started:

[Mumsnet](https://www.mumsnet.com/talk/menopause)

[Menopause Matters](https://www.menopausematters.co.uk/forum/)

[Patient Info](https://patient.info/forums/discuss/browse/menopause-1411)

*Please note these are just a suggestion and are not meant as required list or an endorsement of the content.*

Similar to the podcasts task, could you please keep a log of which you have accessed to help you complete the survey.

All online groups, communities, and forums you access will remain confidential.

At the end of the week, a member of the research team will be in touch via email to ask you to complete an online survey regarding your experience of this task.

**ONLINE GROUPS / FORUMS SURVEY**

**Welcome back!**

Thank you for your continued support for this study.

We hope you have had a chance to interact with online group (s) / forum (s) on menopause. The following pages will ask you to reflect on this experience.

If you have any problems, please get in touch with a member of the research team:

[emails]

What is your participant code?*

*This is the four-character code you were asked to create at the beginning of the study. We suggested your initials and year of birth (e.g., FL70).*

[open answer]

** answer is required for this question.*

Over the last week, how many online groups / forums did you access?

[open answer]

Could you please explain which online group (s) / forum (s) you accessed and why?

[open answer]

Were there any online group (s) / forum (s) that you considered but decided against accessing?

No, Yes

(Selecting yes…) *Please describe your reasons for not accessing.*

How did you engage with the online group (s) / forum (s)?

*Please select all that apply.*

- Browsed / read posts and comments
- Commented on others' posts
- Answered others' questions
- Asked a question
- Created my own post (s)
- 'Liked' posts
- Shared posts
- Other (please describe)

Could you please explain why you engaged with the online group (s) / forum (s) in this way.

[open answer]

What was your experience of accessing online group (s) / forum (s) to gain information on menopause?

*Consider how you engaged with the online group (s) / forum (s), how they made you feel, whether there were any differences between the online group (s) / forum (s)...*

[open answer]

What do you think about the content of the online group (s) / forum (s)?

[open answer]

What was good and bad about the experience?

[open answer]

To what extent do you agree or disagree with the following statements?

|  | Strongly Disagree | Disagree | Neither Agree or Disagree | Agree | Strongly Agree |
| --- | --- | --- | --- | --- | --- |
| I think that I would like to use online group (s) / forum (s) frequently. |  |  |  |  |  |
| I found online group (s) / forum (s) unnecessarily complex. |  |  |  |  |  |
| I thought online group (s) / forum (s) were easy to use. |  |  |  |  |  |
| I think that I would need the support of a technical person to be able to use online group (s) / forum (s). |  |  |  |  |  |
| I found the various functions the online group (s) / forum (s) were well integrated. |  |  |  |  |  |
| I thought there was too much inconsistency in the online group (s) / forum (s). |  |  |  |  |  |
| I would imagine that most people would learn to use online group (s) / forum (s) very quickly. |  |  |  |  |  |
| I found the online group (s) / forum (s) very cumbersome to use. |  |  |  |  |  |
| I felt very confident using the online group (s) / forum (s). |  |  |  |  |  |
| I needed to learn a lot of things before I could get going with the online group (s) / forum (s). |  |  |  |  |  |

[Scored – 1 = Strongly Disagree, 2 = Disagree, 3 = Neither Agree or Disagree, 4 = Agree, 5 = Strongly Agree]

Thinking about accessibility and the technical aspects of online group (s) / forum (s), how did you find engaging with the online group (s) / forum (s)?

[open answer]

Having engaged with online group (s) / forum (s), how knowledgeable do you feel you are about menopause?

No Knowledge, Little Knowledge, Some Knowledge, Knowledgeable, Very Knowledgeable [Likert Scale 1-5]

How much do you agree with the following statement: “I learned something new about the menopause from accessing online group (s) / forum (s)”

Strongly Disagree, Disagree, Neither Agree nor Disagree, Agree, Strongly Agree

[Likert Scale 1-5]

Would you gather information on menopause using online group (s) / forum (s) again?

Yes, No

Could you please explain your answer?

[open answer]

Would you recommend online group (s) / forum (s) to others for health information?

Yes, No

Could you please explain your answer?

[open answer]

What will you take away from the engaging with online group (s) / forum (s) task?

[open answer]

Reflect on your experience using podcasts and rate your agreement with the following statements:

*Likert scale: 1 (Do Not Agree) – 5 (Strongly Agree)*

1. I feel very capable and effective at using online group (s) / forum (s).
2. I feel confident in my ability to use online group (s) / forum (s).
3. Learning how to use online group (s) / forum (s) was difficult.
4. I found the interface and controls confusing.
5. It wasn’t easy to use online group (s) / forum (s).
6. Online group (s) / forum (s) provides me with useful options and choices.
7. I can get the online group (s) / forum (s) to do the things I want it to.
8. I feel pressured by online group (s) / forum (s).
9. Online group (s) / forum (s) feel intrusive.
10. Online group (s) / forum (s) feel controlling.
11. Online group (s) / forum (s) help me to form or sustain relationships that are fulfilling.
12. Online group (s) / forum (s) help me to feel part of a larger community.
13. Online group (s) / forum (s) make me feel connected to other people.
14. I don’t feel close to other users of online group (s) / forum (s).
15. Online group (s) / forum (s) don’t support meaningful connections to others.

**-----End of Survey-----**

[contact information]

**FOCUS GROUP SCHEDULE**

Please think back on the three tasks you completed with the internet, podcasts and online groups.

How did you find this experience?

Focus in on podcasts and online groups

Any preferences for the different platforms? Why would you choose one over the other?

What were the expectations of each of these tasks and how did this compare to the reality?

Good and bad experiences? Any challenges?

Would you continue to use any of these methods in the future and why?

Share top websites, podcasts and groups for discussion.

Websites: NHS (13), Menopause Matters (11), Balance (8). Podcasts: The Dr Louise Newson Podcast (9), The Happy Menopause Podcast (5), Menopause Matters (4). Online Groups: Menopause Matters (10), Mumsnet (6), Patient Info (5).

As you can see, there were similar sources accessed across the tasks. What differences, if any, did you experience in accessing this ‘same’ information through different methods?

Did you seek out the same sources in each task?

What was the impact of using multiple platforms?

Were there any overlaps in information or contradictions? How do you make sense of this?

Credibility of sources/information.

How did you assimilate the information from across platforms?

** Role of Google, ‘people also asked’ section **

Scores on perceived knowledge of menopause, slightly higher after internet and podcasts tasks, similar scores for baseline and after online groups task.

Why do you think this is?

Did your response to the questions regarding your knowledge of menopause reflect new knowledge gained from the task or a confirmation of information you already knew?

What did you get out of each task? Tangible or fluffy

Circling back to the differences in the platforms,

When would you use the internet vs. podcasts vs. online groups?

Needs dependent (information/emotional) or certain points in menopause transition?

Ask participants to draft a ‘timeline’ of access to information – inc. technology and sources

What do you think about digital health information regarding menopause?

**FOLLOW -UP SURVEY**

**Hello again!**

Thank you for your continued support for this study.

If you have any problems, please get in touch with a member of the research team:

[emails]

What is your participant code?*

*This is the four-character code you were asked to create at the beginning of the study. We suggested your initials and year of birth (e.g., FL70).*

[open answer]

** answer is required for this question.*

How knowledgeable do you feel you are about menopause?

No Knowledge, Little Knowledge, Some Knowledge, Knowledgeable, Very Knowledgeable *[Likert Scale 1-5]*

Since attending the focus group, have you looked for digital information on menopause? This could be using the internet, or social media or podcasts.

Yes, No

Since attending the focus group, have you had any communication with a Health Care Professional (e.g., a GP) about your menopause experience or symptoms?

Yes, No

What have you taken away from participating in this research study?

[open answer]

What impact has taking part in this research study had regarding your knowledge and attitude towards menopause?

[open answer]

Which of the following would best describe your engagement with digital technology?

*This could include anything from using the internet to accessing apps or using podcasts.*

1. *Never have, never will*
2. *Was online, but no longer*
3. *Willing and unable*
4. *Reluctantly online*
5. *Learning the ropes*
6. *Task specific*
7. *Basic digital skills*
8. *Confident*
9. *Expert*

*[Scored 1-9]*

Please score yourself on how comfortable/able you are doing the following tasks online.

|  | Can't do/don't know what it is | Would need help to do | Could do with difficulty | Could do | Expert (could teach others) |
| --- | --- | --- | --- | --- | --- |
| Send an email |  |  |  |  |  |
| Delete spam emails |  |  |  |  |  |
| Find stuff using a search engine such as Google |  |  |  |  |  |
| Watch a video on YouTube or iPlayer |  |  |  |  |  |
| Fill out an application form or buy something online |  |  |  |  |  |
| Use a mobile app |  |  |  |  |  |
| Evaluate whether a website is safe/can be trusted |  |  |  |  |  |

*[Scored - 0 = Can't do/don't know what it is, 1 = Would need help to do, 2 = Could do with difficulty, 3 = Could do, 4 = Expert (could teach others)]*

**-----End of Survey-----**

Debrief…
